# Comparative Proteomic Study Reveals the Molecular Aspects of Delayed Ocular Symptoms Induced by Sulfur Mustard

**DOI:** 10.1155/2015/659241

**Published:** 2015-01-21

**Authors:** Zaiddodine Pashandi, Neda Saraygord-Afshari, Hossein Naderi-Manesh, Mostafa Naderi

**Affiliations:** ^1^Department of Biophysics, Faculty of Biology, Tarbiat Modares University, P.O. Box 14115-175, Tehran, Iran; ^2^Department of Ophthalmology, Chemical Research Center, Baqiyatallah University of Medical Sciences, Tehran, Iran

## Abstract

*Objective.* Sulfur mustard (SM) is a highly reactive alkylating agent which produces ocular, respiratory, and skin damages. Eyes are the most sensitive organ to SM due to high intrinsic metabolic and rapid turnover rate of corneal epithelium and aqueous-mucous interfaces of the cornea and conjunctiva. Here we investigate underlying molecular mechanism of SM exposure delayed effects which is still a controversial issue after about 30 years. *Materials and Methods.* Following ethical approval, we have analyzed serum proteome of ten severe SM exposed male patients with delayed eye symptoms with two-dimensional electrophoresis followed by matrix-assisted laser desorption/ionization time-of-flight/time-of-flight mass spectrometry. The western blotting was used to confirm the proteins that have been identified. *Results.* We have identified thirteen proteins including albumin, haptoglobin, and keratin isoforms as well as immunoglobulin kappa chain which showed upregulation while transferrin and alpha 1 antitrypsin revealed downregulation in these patients in comparison with healthy control group. *Conclusions.* Our results elevated participation of free iron circulatory imbalance and local matrix-metalloproteinase activity in development of delayed ocular symptoms induced by SM. It demonstrates that SM induced systemic toxicity leads to some serum protein changes that continually and gradually exacerbate the ocular surface injuries.

## 1. Introduction

Sulfur mustard (2,2′-dichloroethylsulfide; SM) is a bifunctional alkylating and vesicant agent with the highest military significance. After the First World War, Iraq's military usage of SM against Iranian military and civilians as well as their own Kurdish civilians (1980–1988) was the most extensive usage of this agent. The outcome of this genocide was over 100,000 chemical warfare victims that have been grouped into three categories of injuries based on affecting dose and time (mild, moderate, and severe). One-third of these victims are still suffering from SM delayed effects which present an abrupt onset within a period of anywhere from 1 to 40 years after initial exposure [1, 2]. Medical effects of exposure to SM include ocular, skin, and respiratory system damage as the three main areas of concern [1–3]; furthermore several studies indicate agitation impression of SM in gastrointestinal, hematological, neuropsychiatric [4], and immune system effects [5] and skin cancer increasing [6, 7]. Patients with delayed ocular manifestations of SM suffer from itching, photophobia, limbal ischemia, corneal neovascularization, conjunctival, thinning, and irregularity, chronic blepharitis, corneal degeneration, recurring epithelial lesions, meibomian gland dysfunction, and decreased vision [8–10]. It is considerable that there are significant similarities with known pathological effects of radiation-induced damages like skin erythema, mucositis, nausea, and diarrhea for acute effects and fibrosis, organ dysfunction, necrosis, and vascular damages for late effects which are the consequences of an imperfect tissue remodeling [11, 12].

There are several reports on SM effects and its molecular bases in patients. The most considered mechanisms include SM-induced DNA alkylation damage, poly(ADP-ribose)polymerase activation, calcium signaling and calmodulin, nitric oxide signaling and oxidative stress [13], inflammatory reactions, matrix-metalloproteinase activity [14], immunologic effects, neurotrophic effects, and limbal stem cell deficiency [15]. Although the phenomena considered sounds to be similar to the main known mechanism involved in radiation-induced late effects (inflammation, oxidative damages, fibrosis, vascular alterations, and cellular depletion) [11, 16, 17], the underlying pathogenesis and molecular basis of SM acute and delayed effects are still a controversial issue.

Ocular surface damages are primarily due to local contact to SM but its systemic toxicity may lead to some serum protein changes that may continually and gradually exacerbate the ocular surface injuries. Here we have analyzed serum proteome of patients against healthy control groups, by 2DE followed with MALDI TOF/TOF mass spectrometry to identify molecular basis of delayed ocular symptoms induced by severe SM exposure. Furthermore, we have evaluated several-sample processing approach in order to overcome serum proteomic major challenges such as wide dynamic range of protein concentration and presence of high abundance proteins like IgG and albumin.

## 2. Materials and Methods

### 2.1. Serum Sampling

Following institutional ethical approval and written informed consent, blood samples were obtained from ten victims with known pathology of SM severe ocular effects and ten controls by venipuncture into red top tubes (without coagulant activator). All severe victims were man with average age of 54 ± 4.2 in which nearly 28 ± 4.6 years have passed since injury. Some of them were treated corneal transplant surgery and none have any significant pulmonary and skin complications. The blood was centrifuged (for 15 minutes at 1200 g in room temperature) immediately to prevent secretion interference of blood cells during clotting. Obtained plasma was allowed to clot naturally at 4°C less than 60 minutes to prevent protease activities. Then the serum was separated from coagulation factors after centrifugation at 15000 g at 4°C for 15 minutes. Finally, the serum samples then delipidated by centrifuging at 14000 rpm at room temperature for 15 min [18] and stored in −80°C.

### 2.2. Albumin and Immunoglobulin Depletion

#### 2.2.1. 10% Trichloroacetic Acid (TCA)/Acetone

TCA precipitation was carried out as modified method reported by Chen et al. [19]. 25 *μ*l of human serum was precipitated by addition of 100 *μ*l of 10% TCA/acetone solution and was mixed by gentle vortexing. The mixture was incubated at −20°C for 90 minutes and then centrifuged at 15000 g and 4°C for 15 minutes. Then 1 mL of ice-cold acetone (−20°C) was added to wash the precipitate and supernatant was separated. The sample incubated in −20°C for 15 minutes and centrifuged as above. The acetone containing supernatant was removed and the precipitate was dried in air a few minutes.

#### 2.2.2. Chloroform/Methanol

This procedure was carried out as reported by Wessel and Flugge [20]. One volume serum sample (25 *µ*l) was mixed with four-volume methanol. Simultaneously by vortex one-volume chloroform and then three-volume double distillate water (DDW) were added and then centrifuged at 14000 g for 1 min and aqueous layer was removed. Again with vortexing four-volume methanol was added and centrifuged at 14000 g for 2 min. The pellet was washed by cold acetone (−20°C), air-dried for a few minutes, then resolubilized in sample buffer, and concentrated.

#### 2.2.3. Acetone

One-volume serum sample was mixed with four volumes cold acetone (−20°C), incubated for 120 min in −20°C, and centrifuged at 15000 g for 15 min. The pellet was washed by cold acetone, air-dried for a few minutes, and concentrated after resolubilization in sample buffer.

#### 2.2.4. Ammonium Sulfate

One volume of serum was mixed with two volumes of DDW and 0.023 gr ammonium sulfate was added gradually. Incubated about 30 min at room temperature and then centrifuged at 12000 rpm for 10 min. The pellet was washed by cold acetone and air-dried for a few minutes and concentrated after resolubilization in sample buffer.

#### 2.2.5. Acetonitrile (ACN)

The ACN precipitation was carried out as reported by Merrell et al. [21] with minor modification. One volume of serum was mixed by two volumes of pure ACN. They were immediately vortexing for 5 sec vigorously, incubated for 30 min at room temperature, and then centrifuged at 12000 rpm for 10 min. Upper solution was incubated by cold acetone for 60 min and centrifuged as above. The pellet was resolubilized in sample buffer and concentrated.

#### 2.2.6. ProteoMiner Protein Enrichment Kit

One volume of serum sample was performed by ProteoMine Protein Enrichment Kit as manual protocol in four steps: column preparation, binding, washing, and elution. Elution step remained acidic solution, so it was adjusted to pH: 7.4 by PBS buffer; then in order to remove ionic contaminant adjusted to 1 ml by cold acetone and incubated for 90 min. after centrifugation the pellet was air-dried and resolubilized in sample buffer and concentrated.

### 2.3. 2DE

Precipitated proteins obtained from any of the above methods were separately dissolved in sample buffer (sodium dodecyl sulfate (SDS): 2% w/v and dithiothreitol (DTT): 1.25% w/v) and their concentration was determined by UV absorbance at 280 nm (NanoDrop 2000c, Thermo Scientific, NanoDrop Product, Wilmington, DE, USA). Then 80 *μ*g proteins were diluted to 300 *μ*l by rehydration buffer (urea: 7M, thiourea: 2M, CHAPS: 4% w/v, ampholyte 3/10: 2% w/v, DTT: 100 mM). The rehydration stage was performed in 17 cm IPG nonlinear pH: 3–10 strips (Bio-Rad, Hercules, CA, USA) about 16 h actively (constant voltage: 50 V). Immediately isoelectric focusing was performed according to the program: (i) up to 150 V for 90 min rapidly (desalting); (ii) focused at 2000 V for 180 min linearly; (iii) up to 10000 V for 240 min linearly; (iv) finally, maintained at 10000 V for a total of 60000 V-h rapidly. During isoelectric focusing (IEF) we use wicks before desalting (anode and cathode: DDW) and after desalting (anode: DDW, cathode: DTT (20 mM)) step. Then IEF strips were equilibrated for 20 minutes in both equilibration buffer I (urea: 6 M, Tris-Hcl: 1.5 M pH: 8.8, SDS: 2% w/v, glycerol: 20%, DTT: 2.5% w/v) and equilibration buffer II (urea: 6 M, Tris-Hcl: 1.5 M pH: 8.8, SDS: 2% w/v, glycerol: 20%, iodoacetamide: 4% w/v), respectively. The second dimension was performed on 11% SDS polyacrylamide gels at constant current ((i) 16 mA/Gel for 30 min; (ii) 24 mA/Gel) in a Protean II xi 2-D cell (Bio-Rad, Hercules, CA, USA). After protein fixation for overnight with methanol containing acetic acid and formaldehyde, the gel was stained according to MS-compatible silver nitrate procedure according to Yan et al. [22].

### 2.4. Image Processing and Statistical Analysis

After staining gels were scanned by GS-800 Calibrated Densitometer (Bio-Rad, Hercules, CA, USA), the scanned gels were analyzed with SameSpots Progenesis version 3.3 (and 4.1) (Nonlinear Dynamics, Durham NC, USA). The relative intensities of spots were used for comparison between chemical warfare victims and control groups. For a comparison of protein peak intensity differences between two groups, analysis of variance (ANOVA) was performed. The software provides some powerful multivariate statistics analysis including principal component analysis (PCA), correlation analysis, power analysis, and *q*-values (false discovery rate adjusted *P*-values) to make reliable biomarker detection and explore data trends. Also maximum of coefficients of variance (CV%) compared between two groups according to the spots normalized volume to explore specific groups of spots that meet chosen statistical thresholds.

### 2.5. MALDI TOF/TOF and Database Searching

The selected spots were separated from gel manually and MS spectrometry analysis was done at the University of York “Source BioScience LifeSciences, England.” Then identification of protein query was performed by using the MASCOT program searches within “NCBInr 20101130” database according to the following parameters: peptide mass tolerance, 100 ppm; MS/MS ion mass tolerance, 0.5 Da; allowing up to one missed cleavage; variable modifications considered were methionine oxidation and cysteine carboxy-amido-methylation.

### 2.6. Western Blotting

Equal amount of depleted serum of each groups was pooled and then separated by 11% one-dimensional SDS-PAGE and transferred to nitrocellulose membranes in a transblot electrophoresis transfer cell (Bio-Rad, Hercules, CA, USA). Western blot analyses were performed by using rabbit polyclonal antibodies against Tf (diluted 1 : 1000, Abcam) and A1AT (diluted 1 : 500, Sigma-Aldrich). The membranes were blotted with a horseradish peroxidase-conjugated goat anti-rabbit IgG secondary antibody (diluted 1 : 20000, Sigma-Aldrich) for 1 h and then detected with enhanced chemiluminescence reagents for 1 min. Band intensities were quantified using “GelQuant.NET software provided by http://www.biochemlabsolutions.com/.”

## 3. Results

### 3.1. Sample Preparation

**Figure 1:**
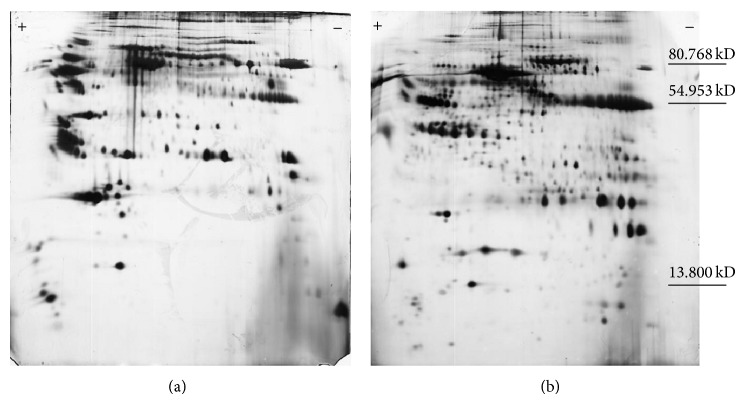
2DE gel comparison: 10% TCA/acetone (b) and ProteoMine large-Capacity Kit (a). Molecular weight assigned by software according to intrinsic standards and verified according to identical proteins in SWISS-2DPAGE database.

Among precipitation methods, 10% TCA/acetone indicates better 2D pattern according to the removal of albumin and IgG (Table 1). Also this precipitation method in comparison with ProteoMiner Kit shows more spot numbers while ProteoMiner Kit removes albumin and IgG almost completely, leading to nonspecific removal of some proteins and minor spot number (Figure 1).

### 3.2. Image Processing and Protein Spots Detection

**Figure 2:**
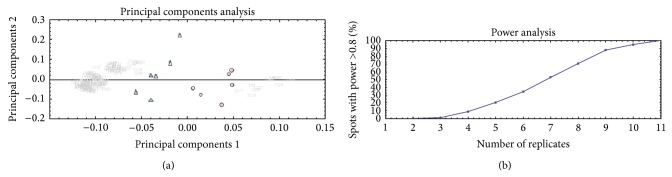
Multivariate statistics. (a) Principal component analysis (PCA) plots for spots with ANOVA *P* value < 0.05. Principal components 1 and 2 are 56.74% and 9.69%, respectively. Control (circle); patient (triangle). (b) Power analysis diagram according to those spots with ANOVA *P* value < 0.05.

**Figure 3:**
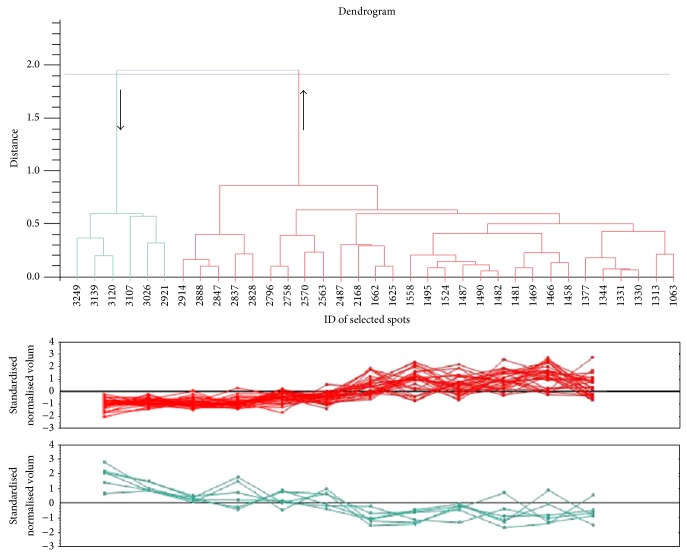
Correlation analysis dendrogram for final selected spots indicates similarity in their expression profiles. Six spots indicated in left of dendrogram showing downregulation in patient while others are upregulated.

The quality of grouping was evaluated by PCA plot for spots with “anova *P* value < 0.05” (Figure 2(a)) as recommended. Comparison of principal components 1 and 2 when all spots were selected (26.86% and 13.66%, resp.) against when only significant spots with *P* value < 0.5 were selected (56.74% and 9.69%, resp.) clearly indicates that the groups are more strongly separated in PCA plot. Analysis of power was performed according to recommended threshold for that, adjusted at power > 0.08, among selected spots with ANOVA *P* value < 0.05 (Figure 2(b)). This diagram indicates at least nine replicates needed to achieving 80% confidence in the experimental data to find the differences that do actually exist. Similarity in expression profile of selected spots was evaluated using correlation analysis (Figure 3). The obtained dendrogram reveals that, except six spots with downregulation characteristic in victims, rest of them represent upregulation variation in expression profile.

Overall, with software analysis 617 spots were detected on silver stained pattern in this study. Among them 102 spots were tagged with ANOVA *P* value < 0.05, 67 spots were tagged with fold ≥2, and 42 spots were tagged with power >0.8. Eventually, result of multivariate statistics leads to selection of twenty spots with both ANOVA *P* value < 0.05 and power ≥ 0.8. In final stage thirteen spots (except keratin 1) were identified by using MALDI TOF/TOF mass spectrometry and search within NCBI database with MASCOT (Table 2).

### 3.3. Validation by Western Blotting

**Figure 4:**
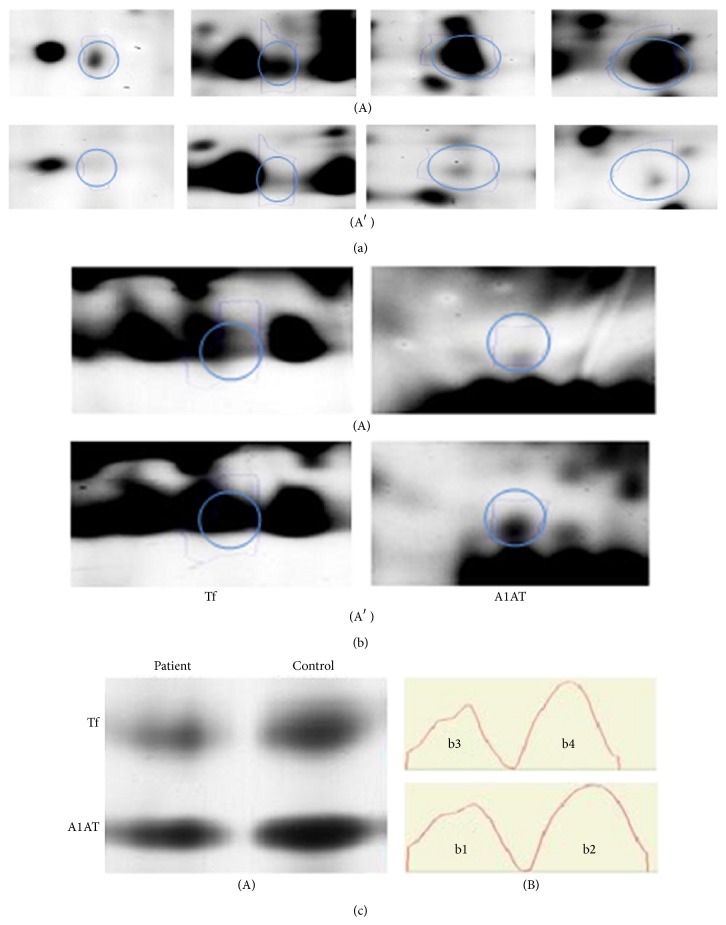
Protein expression profile. (a) Different spots of serum haptoglobin (Hp) isoforms that are identified by MALDI TOF/TOF. Upper (A) and lower (A′) rows refer to patients and controls, respectively. (b) Transferrin (Tf) and alpha 1 antitrypsin (A1AT). Upper (A) and lower (A′) rows refer to patient and control, respectively. (c) (A) Western blot validation of transferrin (Tf) and alpha 1 antitrypsin (A1AT). (B) Intensity profile obtained with* GelQuant.NET software provided by* http://www.biochemlabsolutions.com/. Background signal is 782907.395 and band signals are 1457886.7 in b1, 2030609.6 in b2, 1114966 in b3, and 1534711.7 in b4, respectively.

To confirm the mass spectrometry obtained data, we performed western blot analyses of two proteins Tf and A1AT as shown in Figure 4(c). The immunoblot analysis also showed decreased A1AT and Tf isoforms in the pooled serum of severe patient compared with healthy control group.

## 4. Discussion

According to mentioned approach we identified thirteen proteins including albumin, keratin (except keratin 1), and Hp isoforms as well as immunoglobulin kappa chain that were upregulated in patients plus two downregulated proteins including Tf and A1AT.

### 4.1. Investigation of 2DE Method

Our finding showed that Chen et al.'s [19] procedure eventuates the best quality of two-dimensional gel profile in comparison with relevant available precipitation protocols. In addition, further modification of chen's method indicated more protein spots as well as less streaking in 2DE profile than ProteoMiner protein enrichment Kit (Figure 1). TCA/acetone precipitation has shown good potential for minimizing protein degradation by proteases and removal of contaminant such as salts [23].

### 4.2. Hp

All identified Hp isoforms showed upregulation in our study (Figure 4(a)). Hp is a plasma *α*_2_-glyco-protein that belongs to the family of acute phase proteins [24]. The Hp-1 and Hp-2 have the same *β* chain but they differ in their *α* chain isoforms [25].

There are several well-known roles for Hp, but the more significant of them is as hemoglobin (Hb) binding protein. Their complex serves to prevent the oxidation damages [26, 27]. Iron derived from Hb can catalyze a series of oxidative reaction that is able to participate in organic and inorganic oxygen radical reactions and promoting the accumulation of hydroxyl radicals according to Fenton and Heber-Weiss reactions [28, 29]. Also reactive oxygen and nitrogen spaces seem to contribute as key elements in the radiation-induced late effects which could promote the fibrogenesis through contribution in the transforming growth factor-*β* (TGF*β*) signaling pathway activation [11, 17]. It is notable that the complexes of Hp-2∖Hb have a higher half-life in the circulation than Hp-1∖Hb complexes, causing increased accumulation of Hb in the plasma and the tissues, particularly in the proximal tubule of the kidney, resulting in vascular damages [30]. Hp plays a double-edge role. While its expression is elevated to prevent oxidative stress of Hb derived iron via stabilizing heme in the heme pocket of Hb, increasing of Hp-2 genotype due to longer half-life of its Hp-Hb complexes in circulation leads to increased accumulation of Hb and its derived iron in the plasma and the tissues. Many authors reviewed free iron oxidative effects in human health [31–34]. Also the allele frequencies of Hp and their association in diseases are reviewed in detail by Carter and Worwood [35].

Already Mehrani et al. found Hp isoforms overexpression in plasma of lung disease patient whom exposed by SM [36]. They concluded ongoing tissue remodeling is involved in SM exposed lung damage patients. In addition, they found Hp isoforms in bronchoalveolar lavage fluid of SM exposed patients were significantly elevated in moderate and severe lung disease patients compared to mild and healthy controls [37]. de Kleijn et al. have shown that arterial expressed Hp stimulates angiogenesis, tissue regeneration, and cell migration through inducing accumulation of gelatin by inhibition gelatinases such as MMP-2 and MMP-9 [38]. These collagen deposition and extracellular matrix accumulation have been also due to activation of TGF*β* signaling pathway after radiation exposure [11]. From this point of view our finding could elevate the HP and MMP roles in delayed SM ocular pathology which are also previously discussed by Sardasht-Iran cohort studies in long-term pulmonary complication induced by SM [39, 40].

### 4.3. Tf

In our study Tf has been downregulated (Figure 4(b)). Tf is a plasma glycoprotein which has the capacity to bind to two free atoms of ferric iron (Fe^3+^) in biological fluid [41].

Tf represents a protective mechanism against the presence of free iron in the plasma [42], which could be extremely toxic. The increasing level of serum Tf leads to iron deficiency anemia while decreasing level of that leads to iron overload disorders [31, 43, 44]. The level of Tf also decreases during inflammation, infection, and malignancy conditions [45]. Therefore reduction of Tf level as we found could lead to imbalances in free iron homeostasis effects including free radical production, mutation, and also cell damages. Furthermore, Tf deficiency anemia could induce expression of transcription factors such as hypoxia induced factors due to lack of oxygen. Hypoxia promotes the formation of blood vessels and induces angiogenesis process [46]. This tissue hypoxia could seemingly be one of the mechanisms perpetuating the fibrogenic response in radiation-induced late effects due to vascular damage and tissue remodeling [11, 17].

### 4.4. A1AT

This protein also revealed downregulation in our study (Figure 4(b)) while Yarmohammadi et al. have reported significantly higher amount of salivary A1AT in salivary of sulfur mustard exposed patients 20 years after the exposure [47].

A1AT like Hp is an acute phase glycoprotein and prototypic member of the Serpin family [48, 49]. It has a wide spectrum of antiprotease activity and inhibits several serine proteases such as neutrophil elastase, cathepsin-G, and proteinase-3 [50]. It thought to play an important role in limiting host tissue injury by proteases at sites of inflammation [49]. Several studies have shown that A1AT specifically inhibits the activity of caspase-3 [51–53]. A1AT deficiency is the most widely recognized abnormality of a proteinase inhibitor. The inherited A1AT deficiency exhibits an increased susceptibility to chronic inflammatory conditions, including chronic obstructive lung disease, liver diseases, and occasionally, systemic vasculitis, and necrotizing panniculitis [54]. Here, it appears that lack of A1AT leads to protease induced tissue damages which cooperate with MMP activity eventuating cell apoptosis which could promote in association with free iron toxicity [52, 53, 55].

Findings that A1AT enhances the synthesis of both transferrin receptor and ferritin revealed a role of A1AT in iron metabolism [48]. Here it would be considered maybe that A1AT reduction affects incompetency of Tf receptor and ferritin syntheses and lead to Hp over expression due to cytokines (TNF-*α*) stimulation effects [48, 56, 57]. Also these cytokines showed a significant increase in radiation-induced chronic inflammation [12].

## 5. Conclusions

Our goal was to find a molecular basis for sulfur mustard delayed ocular symptoms and eventually treatment of patients. Finally, our finding elevated important effects of free iron induced oxidative stresses which are supplemented with protease activity (especially MMP) in development of delayed eye symptoms which both indirectly could uphold cell depletion supposition as bases for delayed ocular symptoms. Although the rules of all mentioned proteins are not specified here completely, delayed ocular injuries may be partially explained by these protein changes. Also it can be inferred that changes in serum constituents are due to systemic side effects of SM rather than its local ocular side effects. We hope that our results have revealed some furtive aspects of SM exposed delayed ocular symptoms that will be promoted by future studies.

## Figures and Tables

**Table 1:** Precipitation methods and ProteoMiner protein enrichment kit efficiency.

Precipitation method	Protein amount before precipitation (*μ*g/*μ*L)	Protein amount after precipitation (*μ*g/*μ*L)	Percentage of recovery (%)	Number of spots
Acetone	62.90 ± 3.67	36.96 ± 4.33	58.76 ± 7.68	410 ± 9
ACN	62.90 ± 3.67	44.02 ± 2.01	69.98 ± 5.18	392 ± 11
10% TCA/acetone	62.90 ± 3.67	28.21 ± 3.21	44.85 ± 5.73	438 ± 8
Ammonium sulphate	62.90 ± 3.67	46.47 ± 4.06	73.88 ± 7.66	375 ± 14
Chloroform/methanol	62.90 ± 3.67	50.18 ± 2.67	79.78 ± 6.29	357 ± 9
10% TCA/acetone	62.90 ± 3.67	28.21 ± 3.21	44.85 ± 5.73	617 ± 12^*^
ProteoMiner protein enrichment kit	62.90 ± 3.67	20.49 ± 3.16	32.58 ± 5.36	576 ± 10^*^

Protein concentration was determined by NanoDrop at 280 nm and a number of spots are counted from 7 cm and 17 cm ^*^gels by SameSpots Progenesis version 3.3. The results are obtained from six 7 cm and ten 17 cm gels.

**Table 2:** MALDI TOF/TOF MS spectrometry protein identification in 2DE map of serum.

Protein name	Accession number	Expression level^*^	Biological function	*P* value	*q* value	Power	Fold	Max CV%^†^	MASCOT protein score	Sequence coverage (%)	PI^‡^	Mw (Da)^¶^
Keratin 1	gi∣7331218	↑	Structural proteins	0.00079	0.04	0.99	2	29.8	121	4	8.16	66149
Keratin 10	gi∣186629	↑		0.00079	0.04	0.99	2	29.8	114	5	4.72	39832
Cytokeratin 9	gi∣435476	↑		0.00444	0.09	0.91	1.5	26.34	174	5	5.19	62320
Keratin 1	gi∣7331218	↑		0.00444	0.09	0.91	1.5	26.34	785	16	8.16	66149
Serum albumin	gi∣28592	↑	Molecular carrier and regulator of osmotic pressure in blood	0.00678	0.11	0.86	1.9	36.42	264	6	6.05	71316
Serum albumin	gi∣28592	↑		0.00519	0.12	0.89	2.1	38.32	149	4	6.05	71316
Serum albumin	gi∣28592	↑		0.00284	0.07	0.94	1.8	28.76	308	9	6.05	71316
Serum albumin	gi∣28592	↑		0.00740	0.11	0.85	1.8	31.43	63	2	6.05	71316
Haptoglobin hp2	gi∣223976	↑	Free hemoglobin binding protein in plasma (iron oxidative activity suppressor)	0.00105	0.04	0.98	2.8	44.80	115	6	6.23	42344
Haptoglobin hp2	gi∣223976	↑		0.00372	0.08	0.92	2.3	40.11	70	3	6.23	42344
Haptoglobin hp2	gi∣223976	↑		0.00705	0.11	0.86	3.2	46.62	139	6	6.23	42344
Haptoglobin hp2	gi∣223976	↑		0.00231	0.06	0.95	3.5	79.20	279	11	6.23	42344
Ig kappa	gi∣106529	↑	Immunoglobulin	0.00511	0.10	0.89	1.8	37.77	54	16	5.61	11161
Transferrin	gi∣339469	↓	Iron transport	0.00979	0.13	0.82	1.5	22.25	50	3	8.40	80768
alpha 1 antitrypsin	gi∣177831	↓	protease inhibitor	0.00159	0.05	0.97	1.9	26.26	58	4	4.91	57706

Max CV% is chosen by comparisons of CV% among control and patient groups. ^*^expression level shows protein changes in the patient in comparison with controls. ^†^Maximum of coefficient of variance obtained in comparison between control and patient group. ^‡^Isoelectric point. ^¶^Molecular weight in Dalton.

## Abbreviations

ACN: Acetonitrile
A1AT: Alpha 1 antitrypsin
ANOVA: Analysis of variance
DTT: Dithiothreitol
DDW: Double distillate water
Hp: Haptoglobin
Hb: Hemoglobin
IEF: Isoelectric focusing
MALDI-TOF/TOF: Matrix-assisted laser desorption/ionization time-of-flight/time-of-flight
MMP: Matrix-metalloproteinase
PCA: Principal component analysis
SDS: Sodium dodecyl sulfate
SM: Sulfur mustard
Tf: Transferrin
TGF*β*: Transforming growth factor-*β*
TCA: Trichloroacetic acid
TNF-*α*: Tumor necrosis factor alpha
2DE: Two-dimensional electrophoresis.
